# Does Frequency of Temporomandibular Disorders Pain Influence on Physical and Emotional Function?

**DOI:** 10.1111/joor.70102

**Published:** 2025-11-17

**Authors:** Goli Chamani, Nora Gourie, Zam‐Zam Osman, Petra Lahdo, Golnaz Barjandi, Malin Ernberg

**Affiliations:** ^1^ Department of Dental Medicine Karolinska Institutet Huddinge Sweden; ^2^ Public Dental Health Region Stockholm Stockholm Sweden

**Keywords:** chronic pain, comorbidity, international classification of orofacial pain, psychosocial functioning, quality of life, temporomandibular disorders

## Abstract

**Background:**

Pain frequency is considered an important aspect of pain chronicity, linked to heightened pain and psychosocial distress. In the International Classification of Orofacial Pain (ICOP), temporomandibular disorders (TMD) are classified as frequent (FR, 1–14 days/month) and highly frequent (HF, ≥ 15 days/month), but whether this is clinically relevant is unknown.

**Objectives:**

This retrospective study aimed to examine differences in FR and HF TMD patients regarding demographics, diagnostics, characteristic pain intensity (CPI) and disability (GCPS), jaw function (JFLS), psychosocial variables (depression, anxiety, pain catastrophizing (PCS) and somatic symptoms) and oral‐health‐related quality of life (OHIP‐5).

**Methods:**

Records of 208 patients with either FR or HF TMD pain (ICOP), were included. All patients had been examined according to the Diagnostic Criteria for TMD (DC/TMD) axis I and axis II. Univariate statistics were used to compare FR and HF TMD, and multivariate statistics to identify variables differentiating HF from FR TMD (*p* < 0.05).

**Results:**

Arthralgia and degenerative joint disease were more common in HF TMD compared to FR (*p* = 0.004 and *p* = 0.010). HF TMD had higher CPI (*p* < 0.001), JFLS (*p* < 0.001), PCS (*p* < 0.001) and OHIP‐5 (*p* < 0.001) scores. Higher CPI, GCPS, PCS, JFLS and OHIP‐5 distinguished HF from FR TMD patients (*p* < 0.05).

**Conclusion:**

HF TMD patients showed increased pain intensity, disability, catastrophizing jaw functional limitations, and reduced quality of life distinguishing them from FR TMD patients. This study is the first to demonstrate the clinical relevance of the ICOP frequency‐based classification of TMD, highlighting its importance in the risk assessment and management of these patients.

## Graphical abstract

Higher temporomandibular disorders pain frequency is linked to more severe physical and emotional impairment.

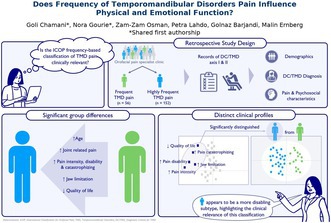

## 1 Introduction

Temporomandibular disorders (TMD) are common chronic pain conditions, affecting approximately 30% of the adult population in Europe with a higher prevalence in women [1, 2]. TMD is characterised by pain and dysfunction in the masticatory muscles and/or temporomandibular joints (TMJ) [3] and is often also associated with chewing difficulties and reduced jaw function [3]. Although some patients have complete remission, the persistence and transition of TMD pain into chronic pain (defined as pain that lasts longer than 3 months) is common. These patients often experience heightened pain, multiple pain sites and comorbid conditions, presenting a clinical challenge for clinicians [4, 5, 6].

Chronic TMD pain is strongly associated with heightened anxiety, depression, stress, reduced quality of life and negative coping mechanisms [7]. These factors can interact in a vicious cycle, where pain increases psychological distress and distress amplifies the pain perception. Oral behaviours (e.g., teeth grinding/clenching), often linked with psychological distress, are common in TMD patients [8], further reinforcing this bidirectional relationship [5, 9, 10]. Despite the availability of standardised diagnostics such as the commonly used Diagnostic Criteria for TMD (DC/TMD), combining clinical examination (axis I) with psychosocial assessment (axis II, questionnaire), treatment outcomes are highly variable [3]. As a result, chronic TMD pain has proven to be a complex condition, becoming a common reason for sick leave with significant burdens on societies and the patients [11, 12].

Pain frequency (i.e., how often pain occurs) is suggested to be an important part of the chronic pain experience [13] and has previously been linked to increased anxiety, depression [14, 15], widespread pain [16] and pain sensitization [17]. In TMD, recent studies have emphasised assessing pain across multiple dimensions such as duration, frequency, intensity and disability, as it may better characterise these patients [18, 19, 20, 21, 22]. As such, higher pain frequency and intensity have been linked to increased pain disability, jaw functional limitation and poorer oral health‐related quality of life [18, 19, 20, 21, 22, 23]. Moreover, longitudinal studies have also found that baseline pain frequency is a predictor of having persistent TMD pain at follow‐up, suggesting a possible risk factor [20, 24]. Assessing pain frequency in clinical practice may thus provide insights into both the severity and prognosis of TMD pain. Accordingly, the International Classification of Orofacial Pain (ICOP) has introduced pain frequency‐based classification of chronic TMD pain i.e., infrequent, frequent (FR, 1–14 days/month) and highly frequent (HF, ≥ 15 days/month) [25]. However, whether this is relevant from a clinical point of view is unknown.

Although previous studies have examined pain frequency in relation to disability and prognosis in TMD [18, 19, 20, 21, 22, 23], no study has applied the ICOP frequency‐based classification to evaluate differences in diagnostic, physical and psychosocial outcomes, limiting our understanding of its impact on TMD pain. The present retrospective study therefore aimed to elucidate this, by examining the distribution of TMD diagnoses, pain intensity, duration and disability as well as jaw function, depression, anxiety, somatic symptoms, pain catastrophizing and oral health‐related quality of life in FR and HF TMD patients. We hypothesised that HF TMD represents a more severe clinical subtype, characterised by increased functional limitation, pain intensity and psychosocial distress compared to FR.

## 2 Methods and Materials

### 2.1 Study Design

This retrospective study examined journal records from patients referred to the specialist clinic for Orofacial Pain and Jaw Function at the University Dental Clinic, Karolinska Institutet (Huddinge, Sweden) between January 1, 2020, and December 31, 2023. A total of 208 patients were included. Because of the retrospective design, no sample size calculation was performed. Instead, all eligible patients within the time frame were included to maximise generalizability.

At their first visit, all patients underwent a standardised DC/TMD [3] clinical examination (Axis I) and completed an extended digital version of the DC/TMD Axis II questionnaire via the secure Research Electronic Data Capture system (REDCap, version 11.1.15) hosted by Karolinska Institutet [26, 27]. The Axis I provided data on TMD pain diagnoses, while the Axis II questionnaire captured demographics (age, birthplace, education level, marital status and occupation), self‐reported Body Mass Index (BMI), pain variables (including questions to diagnose TMD myofascial pain according to the ICOP criteria), and psychosocial status of patients. Patients were subsequently classified as having FR or HF TMD pain according to the ICOP [25]. These two patient groups (i.e., FR and HF TMD pain) were then compared with respect to TMD diagnoses, jaw function, pain characteristics and psychosocial variables.

The selection of patients and methods were approved by the Swedish Ethics Review Authority (Linköping site), 2023‐09‐27 (Reg nr. 2023‐05137‐0).

### 2.2 Participants

Participants between the ages of 16 and 80 were included in this study if they met the ICOP diagnostic criteria for FR or HF chronic TMD pain (duration ≥ 3 months) [25]. To be included, written informed consent was also required agreeing to their journal records being used for research purposes. For those under 18 years of age, consent was obtained from both the adolescent and a parent/legal guardian, in accordance with the ethical approval.

Participants were classified into two groups, as described below.

#### 2.2.1 Frequent TMD Pain

Patients were classified into the FR TMD pain group if they had experienced pain for more than 3 months with ≥ 10 episodes of pain or unremitting pain. These episodes should have occurred once or multiple times a day, each lasting ≥ 30 min and a total daily duration of at least 2 h, on average 1–14 days per month during the past year [25].

#### 2.2.2 Highly Frequent TMD Pain

Patients were classified into the HF TMD group following the same requirements above but with pain occurring on average ≥ 15 days per month during the past year [25].

#### 2.2.3 Exclusion Criteria

Patients were excluded if they did not meet the ICOP criteria for FR or HF TMD pain, had duplicate questionnaires, lacked a clinical examination or patient identification number, reported no pain or a pain duration < 3 months, were younger than 16 years or referred for obstructive sleep apnea. The recruitment process is illustrated in Figure S1.

### 2.3 TMD Pain Diagnosis

Included patients received a TMD pain diagnosis (i.e., myalgia, myofascial pain with referral, arthralgia, headache attributed to TMD, degenerative joint disease and/or disc displacement) based on their DC/TMD Axis I clinical examination. As multiple concurrent diagnoses are possible within the DC/TMD system (e.g., both myofascial and arthralgia), several diagnoses were possible in each patient. DC/TMD examinations were performed by trained, calibrated specialists and residents to ensure diagnostic consistency. Detailed diagnostic criteria for each condition have been described elsewhere [3]. The adult DC/TMD protocol was used for all participants, although not validated for adolescents < 18 years.

### 2.4 Assessment of Pain Variables

#### 2.4.1 Graded Chronic Pain Scale

The Graded Chronic Pain Scale (GCPS) includes three items for pain intensity, three items for pain interference, and one item for the number of days with pain interferences [28]. In this study the validated 1‐month time frame (GCPS v2) was used [29]. The current as well as worst and average pain intensity during the last month are assessed on 0–10 numerical rating scales (NRS) where 0 represents no pain and 10 the worst imaginable pain. The Characteristic Pain Intensity (CPI) (0–100) is calculated as the mean of the three pain ratings multiplied by 10.

The four last items are used for assessing pain interference. A disability score (0–100) is first calculated in a similar manner as CPI from items 5–7 and converted to a 0–3p score (0–29 = 0p, 30–49 = 1p, 50–69 = 2p, 70+ = 3p). The number of days the pain has interfered with daily activities is scored 0–3. The points for the number of days and disability are then added (0–6p). Pain interference is finally graded as Grade 0: None, Grade I: Low intensity pain without disability, Grade II: High intensity pain without interference, Grade III: Moderately limiting disability and Grade IV: Severely limiting disability [30].

#### 2.4.2 Widespread Pain

The Widespread Pain Index (WPI) evaluates pain in 19 specified body locations in five regions: axial (chest, abdomen and neck), upper body right and left side (shoulders, upper arms and lower arms), and lower body right and left side (hips, upper legs and lower legs). The total number of pain locations (0–19) is calculated as the WPI. Scores from 0 to 4 are considered as local pain and scores 5–19 as widespread pain [31].

### 2.5 Assessment of Jaw Function

#### 2.5.1 Parafunctions

The Oral Behaviours Checklist (OBC) enables a simple count to summarise the number of night‐time (two items) and daytime (19 items) parafunctional behaviours [32]. It comprises 21 questions that are scored 0–4 depending on frequency, giving a total score of 0–84. An OBC summary score of 0–16 represents normal behaviours. The instrument is not validated, but a score of 25–62 is reported as a risk for TMD development [33].

#### 2.5.2 Jaw Limitations

Jaw Functional Limitation Scale (JFLS) is a tool used to assess the functional status of the masticatory system in patients with TMD [34]. It measures limitations in three main areas: mastication, vertical jaw mobility, and emotional and verbal expression. It comprises 20 questions, each scored on a 0–10 NRS. A single global score of ‘jaw functional limitation’ can be computed from the available items, where a higher score reflects a more substantial impact on the patient's quality of life.

### 2.6 Assessement of Psychosocial Variables

#### 2.6.1 Depression

The patient History Questionnaire (PHQ‐9) was used to assess the level of depression. It is comprised of 9 items assessing depressed mood. For scoring of each item, a scale of 0–3 is used according to frequency and the total sum of the score (0–27) is calculated. Scores of 5, 10, 15 and 20 represent cut points for mild, moderate, moderately severe and severe depression, respectively [35].

#### 2.6.2 Anxiety

The level of anxiety was assessed with the Generalised Anxiety Disorder (GAD‐7) form. It comprises seven items assessing anxious mood and behaviour, each scored 0–3 according to frequency. A total sum score (0–21) is computed. Scores of 5, 10 and 15 represent cut points for mild, moderate and severe anxiety, respectively [36].

#### 2.6.3 Physical Symptoms

The level of non‐specific physical symptoms was assessed with PHQ‐15. This sub‐scale is comprised of 15 items, each scored 0–2 depending on frequency. A total sum score is computed (0–30) by adding the individual responses. Scores of 5, 10 and 15 represent cut points for low, medium and high levels of physical symptoms, respectively [37].

#### 2.6.4 Pain Catastrophizing

The Pain Catastrophizing Scale (PCS) is a 13‐item self‐report measure of the participants' feelings or thoughts when they are experiencing pain [38]. Each item is scored 0–4. The instrument contains three subscales: rumination, magnification and helplessness. A total score (0–54) can also be calculated. Scores of 0–19 are considered normal, 20–29 are a risk and 30–52 are a high risk of catastrophizing [39].

#### 2.6.5 Distress

The level of distress was assessed with the Perceived Stress Scale (PSS‐10). It comprises 10 questions regarding feelings and thoughts during the last month and measures the degree to which situations in life are appraised as stressful. Each question is scored 0–4 depending on frequency. The total score ranges from 0 to 40. A score from 0 to 12 is considered normal, 13–20 as moderate and 21–40 as severe stress [40].

#### 2.6.6 Sleep Disturbance

Sleep disturbance was assessed with the Insomnia Severity Index (ISI) [41]. ISI comprises seven questions that evaluate sleep quality. Each question is rated on a scale of 0–4, and the total score thus ranges from 0 to 28. A score of 0–7 is considered as no clinically significant insomnia, 8–14 as subthreshold insomnia, 15–21 as clinical insomnia with moderate severity and 22–28 as severe clinical insomnia.

### 2.7 Quality of Life

The Oral Health Impact Profile (OHIP) is a comprehensive, patient‐centred instrument designed to measure the social impact of oral disorders on an individual's well‐being and quality of life [42]. The original version comprises 49 items but there are also shorter versions available. The latest version, OHIP‐5 that was used in this study, consists of five questions related to the experienced frequency limitations regarding teeth, mouth, dentures or jaws in the last month [43] and is recommended for use in orofacial pain [44]. Each question has four alternatives scored 0–4 depending on frequency. The total score (0–20) is calculated as a sum of the five questions. A score of 0–7 is considered as no influence, 8–15 as some and 16–20 as great influence on life quality.

### 2.8 Statistics Analysis

Univariate statistical analyses were performed using Jamovi software (The jamovi project (2025). jamovi (Version 2.6) [Computer Software]. Retrieved from https://www.jamovi.org). The normality of the data was assessed with Kolmogorov–Smirnoff's test. Descriptive data is presented as mean ± standard deviation (SD), median and interquartile range (IQR), or frequency (%), depending on the type of and distribution of data. For group comparison of frequency data, the Chi‐square test was used. Most of the instruments in the axis II questionnaire are composed of several items assessed according to predetermined frequencies, each given an ordinal number. The numbers are then summed up to a total score. We therefore consider parametric statistical analyses not appropriate and used the Mann–Whitney *U*‐test for group comparisons for these data. CPI and WPI were not normally distributed so the Mann–Whitney *U*‐test was used for group comparison of these data as well. However, for age and BMI comparisons were made with the Welsh *t*‐test. A *p*‐value < 0.05 was considered significant.

To assess variables that separate patients in the FR and HF groups, multivariate statistical analysis (MVA) was applied using SIMCA v. 17 (Umetrics, Sartorius Stedim Data Analytics AB, Göttingen, Germany). This approach was adopted due to the high multi‐collinearity of our variables (i.e., that they are intercorrelated), making traditional univariate regression techniques unsuitable. Prior to analysis, all variables were mean‐centred, scaled and if needed, log‐transformed. As the first step in the MVA workflow, unsupervised Principal Component Analysis (PCA) was performed to gain an overview of the dataset, identify underlying patterns and trends and detect possible outliers (that could disturb the subsequent supervised model). In this step, strong outliers were identified using Hotelling *T* ^2^ (a multivariate generalisation of the confidence interval) and moderate outliers were detected by Distance to Model X (DModX). For the objective of group separation, supervised Orthogonal Partial Least Square Discriminant Analysis (OPLS‐DA) was used to analyse group membership (i.e., FR vs. HF TMD pain) using demographic, DC/TMD diagnoses as well as pain, jaw functional and psychosocial variables as regressors (X‐variables). This allowed for the identification of significant predictors (i.e., the X‐variables), while at the same time taking the whole correlation structure into consideration. To analyse correlations within groups between CPI and jaw limitations (JFLS) on one hand, and psychosocial data on the other, OPLS regression was used.

The validity of the OPLS models was evaluated through cross‐validation. For each regression we report the *R* ^2^, *Q* ^2^, Variable Influence of Prediction (VIP) and *p(corr)*. *R* ^2^ describes the goodness of fit and *Q* ^2^ the goodness of prediction. The VIP metric illustrates the importance of each variable in driving the group separation whereas the *p(corr)*, a correlation coefficient, reflects the direction of the relationship and the reliability of each variable's contribution to the model. X‐variables with VIP values > 1.0 and *p(corr)* > 0.4 were considered significant. Lastly, a cross‐validation analysis of variance (CV‐ANOVA) was used to calculate a *p*‐value for each model, determining the validity of the model and the significance (*p* < 0.05) of the observed group separation. For a detailed description of multivariate data analysis, see [45, 46].

## 3 Results

### 3.1 Participants

A total of 727 records were initially screened, from which 519 were excluded due to not meeting the inclusion criteria or insufficient data information in their patient records (Figure S1). The final sample consisted of 208 patients, representing approximately 30% of the potentially eligible patients referred to the clinic during the study period. The sample included 49 (23.6%) men and 159 (76.4%) women (Table 1). These patients were categorised into FR TMD pain (*n* = 56) and HF TMD pain (*n* = 152).

**TABLE 1:** Descriptives characteristic of the 208 patients (49 men and 159 women) included, divided into two groups: High frequent TMD pain and frequent TMD pain.

	Frequent *n* = 56	Highly frequent *n* = 152	*p*
Sex, *n* (%)			0.301
Male	16 (28.6)	33 (21.7)	
Female	40 (71.4)	119 (78.3)	
Age (years), mean (± SD)	32.9 (15.0)	39.1 (17.0)	**0.012**
BMI (kg/m^2^), mean (± SD)	24.4 (6.5)	25.5 (5.2)	0.274
Education, *n* (%)			0.790
Elementary	10 (17.9)	19 (12.5)	
High school	19 (33.9)	50 (32.9)	
University/College	19 (33.9)	53 (34.9)	
Other	2 (3.6)	10 (6.6)	
Unknown	6 (10.7)	20 (13.2)	
Country of birth, *n* (%)			0.443
Sweden	46 (82.1)	112 (73.7)	
Nordic	0 (0)	4 (2.6)	
Europe	2 (3.6)	5 (3.3)	
Other country	8 (14.3)	31 (20.4)	
Employment, *n* (%)			0.630
Employed	27 (48.2)	82 (52.6)	
Unemployed	2 (3.6)	8 (5.3)	
Student	13 (23.2)	27 (17.8)	
Not paid work	2 (3.6)	7 (4.6)	
Sick leave	3 (5.4)	15 (9.9)	
Other	9 (16.1)	15 (9.9)	
Marital status, *n* (%)			0.130
Married	17 (30.4)	68 (44.7)	
Separated	6 (10.7)	12 (7.9)	
Widow	1 (1.8)	3 (2.0)	
Single	8 (14.3)	30 (19.7)	
Other	24 (42.9)	39 (25.7)	

*Note:* Significance indicated with bold figures (Welsh *t*‐test for *p*‐value of age and BMI, descriptives for mean ± SD, or chi‐square test, *p* < 0.05).

Abbreviations: BMI, Body mass index; SD, Standard deviation.

### 3.2 Background Data

Patients in the HF group were significantly older than those in the FR group (*p* < 0.012). Mean BMI was within the normal range (18.5–25 kg/m^2^) in both groups. Approximately two‐thirds of the patients had completed high school or university. Most patients in both groups were born in Sweden and about half of them in both groups were employed. The marital status differed to some extent between groups with a higher percentage of married patients in the HF group. However, apart from age, there were no other significant differences between groups regarding demographic variables.

### 3.3 Pain Variables

The HF group had a significantly higher CPI (median: 47, IQR: 24) compared to the FR group (median: 70, IQR: 23) (*p* < 0.001). Pain interference was also greater in the HF group, with more patients classified in Grades II–IV, whereas most patients in the FR group were classified in Grade I (*p* < 0.001). The WPI in the HF group (median: 5, IQR: 6) did not differ significantly from that in the FR group (median: 4, IQR: 5) (*p* = 0.068).

### 3.4 Jaw Function and Psychosocial Variables

Table 2 shows the scores for jaw function and psychological distress among the groups. Patients in the HF group reported significantly greater jaw functional limitation and pain catastrophizing compared to the FR group (*p* < 0.001). The oral health‐related quality of life was also more impaired in the HF group than in the FR group (*p* < 0.001). There were no significant group differences regarding oral parafunctions, depression, anxiety, physical symptoms, stress or sleep disturbances.

**TABLE 2:** Self‐reported oral parafunctions, jaw functional limitations, and comorbid conditions based on axis II questionnaires in patients with frequent and highly frequent TMD pain.

	Frequent *n* = 56	Highly frequent *n* = 152	*p*
Parafunctions (OBC)	26.0 (12.5)	29.5 (14.0)	0.177
Jaw function (JFLS)	0.9 (1.4)	2.5 (2.5)	**< 0.001**
Quality of life (OHIP‐5)	5.0 (4.3)	8.0 (5.0)	**< 0.001**
Depression (PHQ‐9)	6.0 (8.5)	9.0 (10.0)	0.104
Anxiety (GAD‐7)	4.0 (6.5)	6.0 (9.0)	0.174
Physical symptoms (PHQ‐15)	9.0 (7.5)	10.0 (8.0)	0.052
Pain catastrophizing (PCS)	8.0 (13.5)	19.0 (21.0)	**< 0.001**
Stress (PSS‐10)	11.0 (14.5)	14.0 (16.0)	0.221
Sleep disturbance (ISI)	10.5 (8.0)	11.0 (11.0)	0.537

*Note:* Significance indicated with bold figures (Mann–Whitney *U*‐test for *p*‐value < 0.05). Data show median (IQR) total score.

Abbreviations: GAD, generalised anxiety disorder; ISI, Insomnia severity index; JFLS, jaw functional limitation scale; OBC, oral behaviour checklist; OHIP, oral health impact profile; PCS, pain catastrophizing scale; PHQ, patient health questionnaire; PSS, perceived stress scale.

### 3.5 TMD Pain Diagnoses

Myofascial pain was the most common diagnosis among the patients in both groups affecting 96.2% of the total sample. TMJ pain was the second most common diagnosis (70.7%), followed by headache attributed to TMD (54.8%). TMJ pain and DJD were more prevalent in the HF group compared to the FR group (*p* = 0.004 and *p* = 0.010, respectively). There were no other significant group differences regarding the prevalence of DC/TMD diagnoses (Table 3).

**TABLE 3:** Diagnoses according to the diagnostic criteria for TMD (DC/TMD) and pain interference grade (GCPD) in patients with frequent and highly frequent TMD pain.

	Frequent *n* = 56	Highly frequent *n* = 152	*p*
Chronic myofascial pains			0.630
Myofascial pain	30 (53.6)	73 (48.0)	
Myofascial pain with referral	25 (44.6)	73 (48.0)	
TMJ pain	31 (55.4)	115 (75.7)	**0.004**
Myofascial pain + arthralgia	30 (53.6)	109 (71.7)	**0.015**
H‐TMD	35 (62.5)	79 (52.3)	0.191
DDwR	12 (21.4)	37 (24.3)	0.661
DDwR with intermittent locking	8 (14.3)	18 (11.8)	0.636
DDwoR with limited opening	0 (0.0)	8 (5.3)	0.080
DDwoR without limited opening	2 (3.6)	9 (5.9)	0.502
DJD	9 (16.1)	52 (34.2)	**0.010**
Pain interference			**< 0.001**
Grade I	28 (50.0)	16 (10.5)	
Grade II	18 (32.2)	74 (48.7)	
Grade III	4 (7.1)	26 (17.1)	
Grade IV	2 (3.6)	25 (16.4)	
MD	4 (7.1)	11 (7.2)	

*Note:* Several diagnoses were possible in each patient. Significance indicated with bold figures (Chi‐square test, *p* < 0.05). Data show number (%).

Abbreviations: ACR, American College of Rheumatology; DC/TMD, diagnostic criteria for TMD; DDwoR with limited opening, disc displacement without reduction with limited opening; DDwoR without limited opening, disc displacement without reduction with limited opening; DDwR with intermittent locking, disc displacement with reduction and with intermittent locking; DDwR, disc displacement with reduction; DJD, degenerative disorder; GCPS, graded chronic pain scale; H‐TMD, Headache attributed to TMD; IBS, Irritable bowel syndrome; MD, missing data; TMD, temporomandibular disorders.

### 3.6 Multivariate Analysis

#### 3.6.1 Principal Component Analysis (PCA)

As mentioned, the initial multivariate PCA was performed for overall data overview and to detect outliers. This analysis revealed one strong outlier, identified as deviating from the normal correlation structure using Hotelling's *T* ^2^. This patient was subsequently excluded from further analyses leaving a total of 207 patients. This model had one principal component, with a goodness of fit *R* ^2^ = 0.2 and a predictive ability of *Q* ^2^ = 0.142.

#### 3.6.2 Group Classification and Identification of Discriminant Variables (OPLS‐DA Analysis)

**FIGURE 1:**
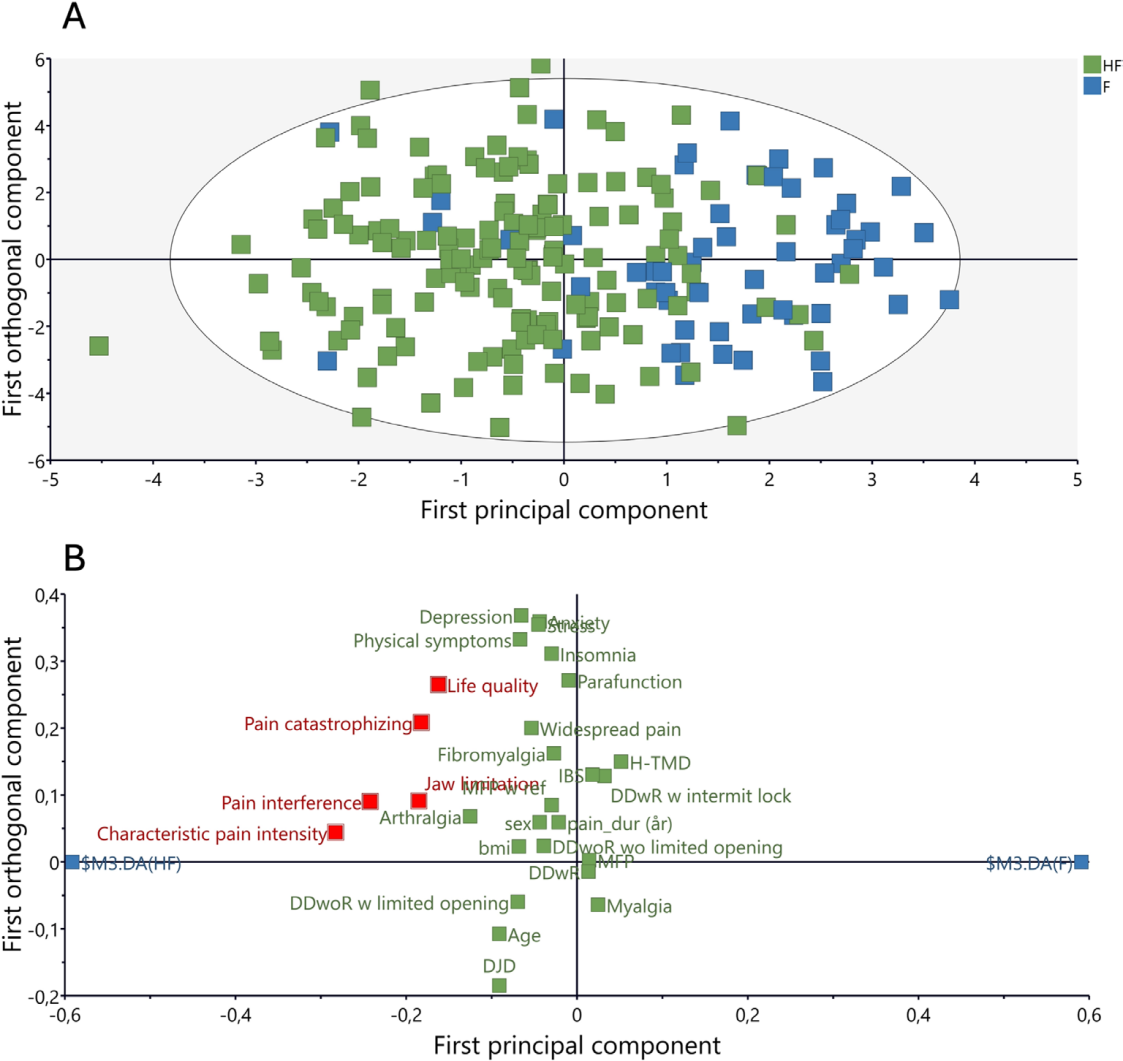
(A) Score plot of the orthogonal partial least square discriminant analysis (OPLS‐DA) model showing the separation between patients with highly frequent (HF; green squares) and frequent (F; blue squares) temporomandibular disorder (TMD) pain. The two axes represent the principal components of the model. Class separation between groups occurs along the principal component axis (inter‐class variation), whereas the orthogonal axis represents intra‐class variation. (B) OPLS‐DA loading plot showing the variables that separated the groups. Red squares show variables that significantly differed. Variables located closer to the group (blue squares) are positively associated to that group.

For the objective of group separation, supervised OPLS‐DA was applied. OPLS‐DA separates the variance in the predictors (X‐variables) into two parts i.e., the predictive of the group classification (Y‐variable) and the orthogonal (uncorrelated) to the Y‐variable. The OPLS‐DA identified one highly significant model (CV‐ANOVA *p* = 1.13177e−09) separating the groups (Figure 1A). This model had 2 principal components and the overall model performance metrics were *R* ^2^ = 0.255 (i.e., the degree of fit to the outcome variable Y) and *Q* ^2^ = 0.217 (i.e., the predictive reliability). The relatively small difference between *R* ^2^ and *Q* ^2^ suggests that the model is reliable and not overfitted. Using a cut‐off of VIP > 1.0 and *p(corr)* > 0.4, five significant variables were identified as responsible for this observed group separation i.e., CPI, GCPS, PCS, OHIP‐5 and JFLS (Table 4 and Figure 1B). In other words, the OPLS successfully separated HF TMD patients from the FR patients with increased pain intensity, interference and catastrophizing together with reduced oral health‐related quality of life and jaw function significantly distinguishing the two groups. Moreover, the strong *p*‐value indicates that the model's ability to separate HF from FR TMD patients using the significant variables is reliable when tested on unseen data (via cross‐validation).

**TABLE 4:** OPLS‐DA analysis showing predictors separating patients with highly frequent (HF; *n* = 149) and frequent (F; *n* = 58) temporomandibular disorders (TMD) pain.

	VIP	*p(corr)*
Characteristic pain intensity (CPI)	2.233	0.808
Pain interference (GCPS)	1.927	0.694
Pain catastrophizing (PCS)	1.569	0.497
Life quality (OHIP‐5)	1.513	0.444
Jaw limitation (JFLS)	1.484	0.525

*Note:* The model had two components and was highly significant (CV‐ANOVA; *p* < 0.001).

Abbreviations: CPI, characteristic pain intensity; GCPS, graded chronic pain scale; JFLS, jaw functional limitation scale; OHIP, oral health impact profile; *p(corr)*, correlation coefficient (−1 to +1); PCS, pain catastrophizing scale; VIP, variable important prediction.

### 3.7 Within‐Group Correlation Analysis (OPLS Analysis)

As both CPI and jaw limitation (JFLS) were identified as significant predictors in the OPLS‐DA, subsequent OPLS regression models were built within each group (i.e., HF and FR TMD pain separately) to specifically assess the correlations between these clinical variables and psychosocial variables. As can be seen in Table 5 significant correlations were found between CPI, pain interference, and pain catastrophizing in the HF group and between CPI, pain interference, pain catastrophizing and life quality in the FR group. In the HF group jaw limitations were significantly correlated to depression, anxiety, pain catastrophizing, stress, insomnia and life quality. For the FR group, the OPLS model was not significant (CV‐ANOVA > 0.05); thus, correlations are not shown.

**TABLE 5:** Correlation coefficients *p(corr)* from OPLS analysis using characteristic pain intensity (CPI) and jaw functional limitations (JFLS) as regressors in 207 patients with highly frequent and frequent temporomandibular disorders pain.

	Highly frequent		Frequent
	CPI	JFLS	CPI
Age	−0.380	−0.352	0.924
BMI	−0.231	−0.012	0.485
Pain intensity (CPI)	—	0.410	—
Pain duration	−0.333	−0.081	−0.335
Widespread pain (WPI)	−0.400	0.166	0.271
Pain interference (GCPD)	**0.827**	0.532	**0.924**
Pain catastrophizing (PCS)	**0.602**	**0.733**	**0.530**
Depression (PHQ‐9)	0.248	**0.825**	0.277
Anxiety (GAD‐7)	0.181	**0.801**	0.324
Physical symptoms (PHQ‐15)	−0.172	0.521	0.321
Stress (PSS‐10)	0.007	**0.716**	0.339
Insomnia (ISI)	0.060	**0.649**	0.159
Parafunctions (OBC)	−0.007	0.458	0.145
Jaw limitations (JFLS)	0.410	—	0.454
Life quality (OHIP‐5)	0.368	**0.722**	**0.475**

*Note:* The OPLS regressions were significant for CPI and JFLS in the Highly Frequent pain group (CV‐ANOVA; *p* < 0.001 and *p* < 0.01, respectively) and for CPI in the Frequent Pain group (*p* < 0.001). Correlations are significant (bold font) when VIP (not shown) is > 1 and *p(corr)* > 0.4.

Abbreviations: CPI, characteristic pain intensity; GAD, General Anxiety Disorder; GCPS, Graded Chronic Pains Scale; ISI, Insomnia Severity Scale; JFLS, Jaw Functional Limitation Scale; OBC, oral behaviour checklist; OHIP, oral health impact profile; OPLS, Orthogonal Partial Least Square; *p(corr)*, correlation coefficient; PCS, Pain Catastrophizing Scale; PHQ, patient health questionnaire; PSS, Perceived Stress Scale; TMD, temporomandibular disorders; WPI, Widespread Pain Index.

## 4 Discussion

The present retrospective study examined the clinical relevance of the ICOP classification of TMD pain into FR (i.e., pain 1–14 days/month for > 3 months) and HF (i.e., pain ≥ 15 days/month for > 3 months) groups by comparing demographics, diagnostic distribution, pain characteristics, jaw function, psychosocial distress and oral health‐related quality of life between the two patient groups. We found that patients with HF TMD pain were significantly older, presented more often with TMJ pain and DJD and had higher CPI, pain‐related disability, PCS, JFLS and OHIP‐5 compared to FR patients. Interestingly, the multivariate analysis confirmed that increased higher CPI, pain‐related disability, PCS, JFLS and OHIP‐5 were significant variables distinguishing HF from FR TMD patients.

Thus, pain frequency seems to be more than solely a descriptor of symptom occurrence, supporting our hypothesis that HF TMD represents a more severe form, characterised by increased clinical burden in terms of pain, functional limitation and catastrophizing.

### 4.1 Distribution of DC/TMD Diagnoses

Both FR and HF patients were mostly diagnosed with myofascial pain, which is consistent with previous research highlighting the predominance of muscle‐related pain in TMD populations [47, 48, 49]. TMJ pain and DJD were significantly more common among HF patients, which could suggest that joint pain may contribute to more frequent pain [49, 50, 51]. On the other hand, the HF group also more frequently had a combination of myofascial pain and arthralgia, which perhaps could indicate that the higher frequency of TMJ pain in HF more reflects an ‘unspecific’ pain spread than TMJ pathology. However, these differences were not significant in the multivariate analysis when also accounting for pain‐related and psychosocial variables. Thus, although TMJ‐related pain is more common in the HF TMD pain group, their clinical experience may be better explained by pain intensity, disability and coping style.

### 4.2 Pain Intensity and Disability

HF patients reported significantly higher CPI and were more often found in the higher disability grades of the GCPS. Multivariate analysis further confirmed that both CPI and GCPS group were significant variables distinguishing HF from FR TMD patients. This interrelationship between pain frequency, intensity and disability is consistent with previous findings. For instance, Salbego et al. (2025) demonstrated that increased pain frequency and intensity were significantly associated with higher GCPS grades [18]. Longitudinal studies have further supported this relationship as Velly et al. (2022) demonstrated that higher CPI, GCPS groups and pain frequency (defined as ‘pain always present’) at baseline predicted persistent pain at 3‐month follow‐up [20], and Rammelsberg et al. (2003) found that baseline pain frequency was the strongest predictor of persistent myofascial pain at 5‐year follow‐up [24]. Similar patterns have been found in headache disorders, where increasing headache frequency has been suggested as a central driver of chronification and central sensitization [17, 52]. Taken together, these parallels suggest that higher TMD pain frequency could indicate chronification. However, this interpretation has been debated, as a previous critical review found no significant differences in pain frequency between acute and chronic TMD pain [53]. It is plausible that the interaction of factors (as illustrated in our multivariate analysis), rather than frequency alone, indicates a more severe clinical presentation potentially leading to chronification. Future longitudinal studies are needed to clarify these relationships.

While HF patients also reported pain in more locations (more widespread pain), the difference from FR patients was not significant (*p* = 0.06). This contrasts with previous research linking widespread pain and referred pain to higher pain frequency [19] and intensity as well as poorer function compared to those with local or regional pain [16]. The reasons for these contrasting results are unclear. On the one hand, it suggests that in this TMD cohort, other factors may be more relevant indicators of pain frequency. On the other hand, methodological variations, cohort characteristics and sample size may also explain these differences.

### 4.3 Physical Function and Oral Health Impact

HF patients, compared to the FR pain group, reported significantly increased jaw functional limitation on the JFLS and more impaired oral health‐related quality of life on the OHIP. These findings demonstrate that frequent pain episodes compromise essential oral functions (e.g., chewing speaking) reducing daily life quality, further highlighting the disabling effect of high‐frequency pain. Previously, the increased frequency of headaches in TMD patients has been linked to increased jaw functional limitations and oral health‐related quality of life [23]. Moreover, both jaw functional limitations and reduced oral health‐related quality of life have consistently been associated with TMD pain [54, 55, 56, 57, 58]. Building on previous research, our findings further demonstrate that these limitations may not be equally distributed among chronic TMD pain patients, but are more common in HF pain, highlighting the impact of pain frequency on these measures and its importance in clinical assessment.

### 4.4 Psychosocial Burden and Pain Catastrophizing

HF patients reported significantly higher pain catastrophizing compared to FR patients, whereas no group differences were found for depression, anxiety, stress, somatic symptoms or sleep disturbances. Pain catastrophizing is a well established predictor of pain intensity, disability and poor treatment outcomes in chronic pain conditions [59, 60, 61], and in our multivariate analysis it was also identified as a significant discriminator between HF and FR groups, highlighting its clinical relevance in frequency‐based classification. However, the absence of group differences in depression and anxiety contrasts with previous studies reporting associations with pain frequency [15, 23]. These differences may reflect methodological differences, different patient populations or limited sample size.

On the other hand, the differences in our results may also be explained by the close interrelationship between catastrophizing, depression and anxiety, specifically since studies attempting to separate their unique contributions have reported mixed findings [62, 63]. For instance, Kjogx et al. (2014) [63] showed that catastrophizing was significantly related to pain only when the analyses were not adjusted for depression and anxiety. Interestingly, however, when pain frequency was considered in their analysis, pain catastrophizing remained significantly related to pain, independent of anxiety and depression, and this moderating effect was only found in cases of high pain frequency, and not in those with low pain frequency [63]. Together with our results, this suggests that pain‐specific negative cognitive processes (such as pain catastrophizing) are more closely linked to pain frequency than generalised psychological distress [63].

### 4.5 Multivariate Analysis and Intercorrelations

The OPLS‐DA identified CPI, pain interference, JFLS, PCS and OHIP as significant variables distinguishing HF from FR patients, suggesting that frequency‐related severity is better characterised by pain intensity, functional limitation and negative cognitive processes rather than demographics (such as age, education, marital status, BMI, etc.), generalised psychological distress or diagnostic differences. Although the explained variation in the OPLS‐DA analysis was moderate, the stability of the OPLS‐DA model and its high statistical significance confirm that these variables reliably drive the observed separation between HF and FR TMD patients. Moreover, our multivariate correlation analysis further demonstrated that JFLS was significantly associated with multiple indicators of psychological distress and reduced quality of life in those with HF but not in FR TMD pain, further suggesting that the psychosocial burden of TMD pain becomes more integrated with functional impairment at higher pain frequencies.

The stronger impact of these variables at higher TMD pain frequency supports our hypothesis that frequency reflects more than symptom occurrence, signalling increased clinical burden and functional impairment. While the importance of capturing this burden is reflected in the assessment within both ICOP [25] and DC/TMD [3], our data extends them by showing that these variables may be more strongly integrated at higher pain frequencies. Our findings further suggest that HF pain may be a more demanding state than FR pain where the interaction of pain intensity, pain interference, jaw limitations and pain catastrophizing reinforce one another. However, it is important to note that although these multivariate statistical approaches identify correlation and interaction between variables, they cannot establish causality, highlighting the need for future studies using mediation analyses to help clarify causal pathways.

### 4.6 Study Strengths and Clinical Implications

This is the first study to systematically apply the ICOP frequency‐based classification (FR vs. HS) to a clinical TMD cohort. A major study strength is the use of standardised diagnostic criteria (ICOP and DC/TMD) combined with validated psychosocial instruments and advanced multivariate statistics. This approach allowed us to assess the complex interrelationship of pain, function and psychosocial factors, thereby indicating HF TMD as clinically different from FR TMD patients while simultaneously identifying the specific variables driving this differentiation.

Thus, the findings of this study validate the frequency‐based classification presented in the ICOP and further highlight the importance of incorporating it in clinical assessments of TMD patients, as it may better reflect the burden of pain. Identifying HF TMD patients may help clinicians in their risk assessment and treatment planning, as these patients may require multidisciplinary management strategies. For instance, combining standard TMD interventions (addressing pain and jaw function) with targeted psychological support to reduce catastrophizing may improve outcomes for these patients. Finally, as HF appears to represent a more difficult condition and reductions in pain frequency have previously been found to improve prognosis, tracking pain frequency in clinical practice may help clinicians evaluate treatment outcomes.

### 4.7 Limitations and Future Directions

However, there are several limitations to this study that need to be acknowledged. First, only patients with complete ICOP and DC/TMD data were included, resulting in 208 participants, which represents approximately 42% of the eligible patients referred during the study period limiting the generalizability of our findings. The retrospective study design also introduces potential biases. For instance, the use of self‐reported questionnaires raises a concern for recall bias whereas the missing data in the self‐reported questionnaires may have led to selection bias as several patients had to be excluded. Additionally, a priori power analysis was not performed due to our study design, which may have limited statistical power. Moreover, medical histories, comorbidities, medication use, and concurrent treatment were considered, which could influence our results. Another limitation is that most Axis II instruments are not validated for use in adolescents < 18 years why data for the patients 16–18 years should be interpreted with caution. Lastly, the use of OHIP‐5 instead of the condition‐specific OHIP‐TMD is a limitation of this study, as it may miss aspects of the quality of life specific to TMD. As such, future studies are needed to validate our findings in larger cohorts, also examining whether the presence of HF TMD pain predicts poorer long‐term outcomes, as suggested by previous longitudinal studies. Moreover, mechanistic research may help determine whether repeated nociceptive input associated with high‐frequency pain promotes central sensitization in TMD (as observed in headache disorders) [52, 63]. Finally, intervention studies may further explore whether targeting catastrophizing and functional limitation specifically benefits HF TMD patients.

## 5 Conclusion

Our findings suggest that pain frequency is a clinically important factor in TMD pain. Patients with HF TMD pain differed significantly from those with FR pain, reporting higher pain intensity, disability, jaw functional limitation, pain catastrophizing and reduced oral health‐related quality of life. These results support the ICOP frequency‐based classification of TMD, confirming our hypothesis that HF TMD pain may reflect a more disabling subtype and thus support its clinical relevance in assessment and management.

## Author Contributions

Malin Ernberg conceptualised the study. Goli Chamani, Nora Gourie, Golnaz Barjandi, Zam‐Zam Osman and Petra Lahdo contributed to data collection. Nora Gourie and Malin Ernberg performed the statistical analysis. Goli Chamani and Nora Gourie drafted the manuscript. Golnaz Barjandi and Malin Ernberg reviewed and edited the manuscript. Malin Ernberg supervised the entire research process. All authors critically reviewed and approved the final version of the manuscript.

## Conflicts of Interest

The authors declare no conflicts of interest.

## Supporting information


**Figure S1:** joor70102‐sup‐0001‐FigureS1.docx.

## Data Availability

The data that support the findings of this study are available from the corresponding author upon reasonable request. The dataset includes patient information from the clinical records of the Orofacial Pain and Jaw Function Clinic, Karolinska Institutet, and cannot be made publicly available due to ethical and privacy restrictions.
