# Supplementary material for: Does Frequency of Temporomandibular Disorders Pain Influence on Physical and Emotional Function?
Source: J Oral Rehabil. 2025 Nov 17;53(2):556–67. doi: 10.1111/joor.70102 (PMC12813525; doi:10.1111/joor.70102)
Supplement: Supplementary file 1 — Figure S1: joor70102‐sup‐0001‐FigureS1.docx. [file JOOR-53-556-s001.docx]

**
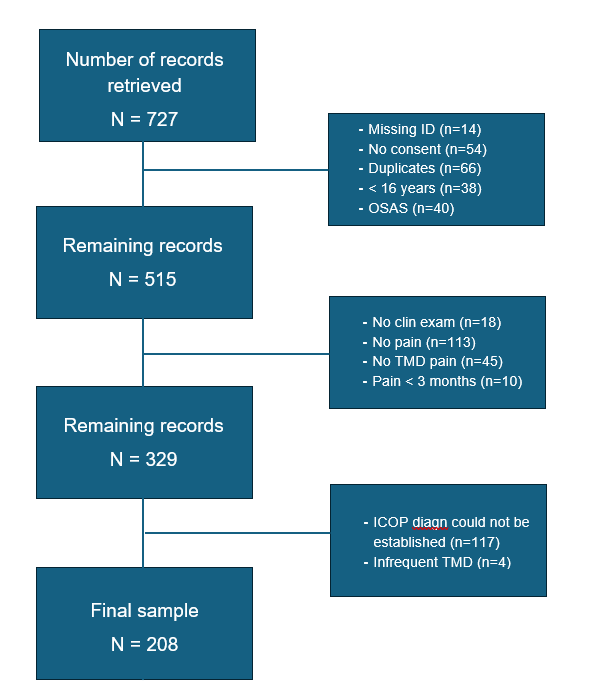
**

**Suppl Fig 1**. Flow chart showing the data-selection process in the study. OSAS: Obstructive Sleep Apnea Syndrome; TMD: temporomandibular disorders; ICOP: International Classification for Orofacial Pain.
